# A syringe method for esophageal Lugol’s iodine chromoendoscopy

**DOI:** 10.1055/a-2213-1316

**Published:** 2023-12-21

**Authors:** Kai Liu, Jiawei Bai, Li Gao, Xin Dong, Ying Han, Zhiguo Liu

**Affiliations:** 1 66352 Xijing Hospital of Digestive Diseases, Air Force Medical University (Fourth Military Medical University), Xi’an, China

Lugol’s iodine chromoendoscopy is the method commonly used to detect and diagnose esophageal squamous cell carcinoma 1 2 . The conventional method of spraying iodine requires iodine solution diluted from the stock solution, a spraying catheter, and cooperation between the endoscopist and the assistant. Here, we report a novel syringe method for iodine spraying without the above requirements.

Before chromoendoscopy, a 20-ml syringe was employed to draw 3 ml of 5% iodine stock solution and 17 ml of air. When staining, the iodine was quickly expelled into the esophagus through the endoscope’s working channel at 20 cm from the incisors ( Fig. 1 ). An iodine mist was created from the rapid spurt of the iodine solution and air, which prompts even distribution of the iodine solution on the esophageal wall ( Fig. 2 **a,b** ). The lower segments of the esophageal wall were stained with the remaining iodine solution in the working channel by repeatedly spraying the air/iodine mixture using the syringe, resulting in consistent and uniform staining of the whole esophagus ( Video 1 ).

**Fig. 1:**
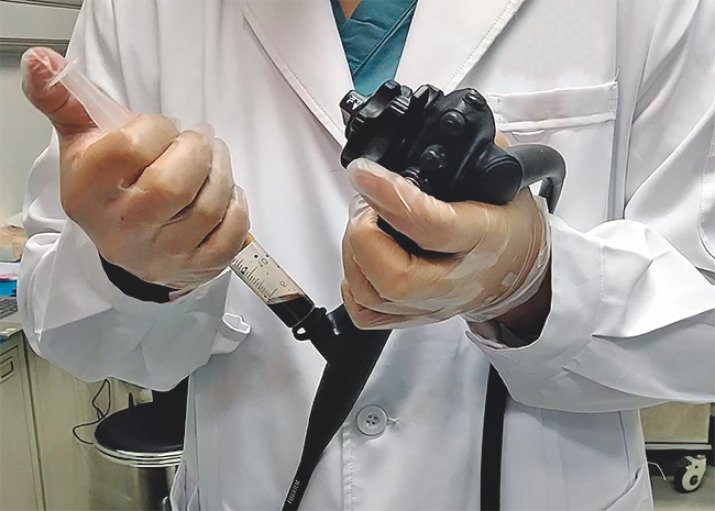
A 20-ml syringe with 3 ml of 5% iodine stock solution and 17 ml of air was employed to spray the iodine solution through the endoscope’s working channel.

**Fig. 2:**
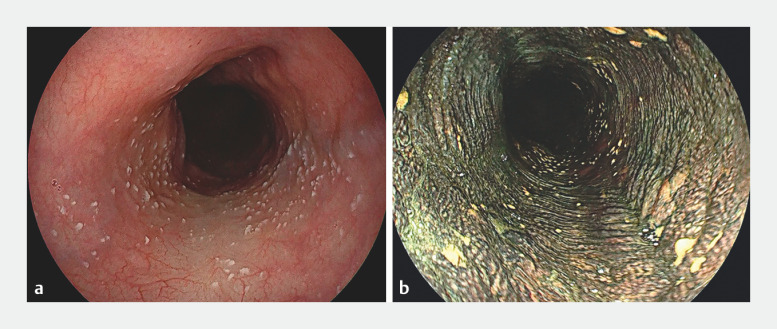
Endoscopic images. **a** Before iodine staining. **b** After iodine staining.

The syringe method for Lugol’s iodine chromoendoscopy.Video 1

The spraying method is safe and effective. Although the iodine mist was created during the chromoendoscopy, the direction of the iodine spray is from the proximal to the distal esophagus and uses only 3 ml of iodine solution. Therefore, theoretically, the incidence rate of coughing/irritation to the larynx caused by the reflux of iodine solution is very low. Our center has used the syringe method in over 100 cases; only one patient experienced coughing. Compared to the conventional method, the syringe method has the following advantages: a single individual method eliminating the need for an assistant; cost-saving with no need for a spraying catheter 3 ; no need to dilute the iodine solution; and reduced flow of iodine into the stomach, which may potentially minimize mucosal injury.

Endoscopy_UCTN_Code_TTT_1AO_2AM

## Notes

Endoscopy E-Videos https://eref.thieme.de/e-videos
*E-Videos* is an open access online section of the journal *Endoscopy* , reporting on interesting cases and new techniques in gastroenterological endoscopy. All papers include a high-quality video and are published with a Creative Commons CC-BY license. Endoscopy E-Videos qualify for HINARI discounts and waivers and eligibility is automatically checked during the submission process. We grant 100% waivers to articles whose corresponding authors are based in Group A countries and 50% waivers to those who are based in Group B countries as classified by Research4Life (see: https://www.research4life.org/access/eligibility/ ). This section has its own submission website at https://mc.manuscriptcentral.com/e-videos .
